# New York Odontological Society

**Published:** 1892-04

**Authors:** 

**Affiliations:** New York Odontological Society


### Reports of Society Meetings.

### NEW YORK ODONTOLOGICAL SOCIETY.

A REGULAR meeting of the New York Odontological Society was held on January 19, 1892, at the New York Academy of Medicine, No. 17 West Forty-third Street, with Vice-President Dr. Brockway in the chair. The secretary read the minutes of the previous meeting, which were approved.

On motion the usual order of business was dispensed with, and the paper of the evening taken up.

Dr. J. V. Porter then read the following paper, entitled, "Pulpless Teeth; Abscess; Treatment, especially Surgical Treatment."

(For Dr. Porter's paper, see page 255).

### DISCUSSION.

*Dr. J. N. Farrar.*—The essayist's paper has in it so much in harmony with my own views that there is not much room to discuss it. I use non-escharotic creosote in the treatment of pulpless teeth that are not associated with abscesses,—I mean wood creosote in combination with alcohol. The object of the alcohol is to enable the creosote to act in the presence of aqueous moisture, if there should be any in the tooth.

The plan of drying teeth with hot air is excellent, but I think that bibulous paper will extract about as much water as the heating process, unless this is kept up for a long time. I do not regard moisture in the dentine as injurious to the tooth, but the pulp-canal should be dry. If there are no abscesses, I do exactly the same as the essayist mentioned,—fill the canal at the first sitting, if I have time. If I do not have that, I close it tightly. The great trouble with many dentists is that they fill temporarily with cotton saturated with some kind of drug, and then do not seal tight the canal. It should be sealed with something that will prevent any kind of micro-organisms from entering. I use phosphate of zinc cement over the cotton. When permanently filling a pulp-canal, I generally plug the apex with gold. What is needed is a small wad of indestructible substance. In early practice I did the same occasionally, but generally I used creosoted cotton, but I had not been in practice long before I found that some of my own as well as other people's cases would begin to annoy the patients after a while. Upon removing the filling of several, it was found that this cotton underwent decomposition by being soaked with moisture, entering it through the foramen. This decomposition was what made the mischief. Then I saw more clearly than ever the value of the use of indestructible material at the apex. Gutta-percha has the same fault as cotton. It being a vegetable substance, it decays in time at the apex of the root. There are many cases, however, in which those materials never cause trouble. But any vegetable matter is liable to decompose, and bring about irritation of the socket. If the canal is perfectly clean, and the apex is properly filled with something that is indestructible, and the main canal filled with phosphate of zinc or gutta-percha, then there is not much liability to future difficulty.

An abscess at the apex of the root is, in one sense, nothing more than a deep-seated boil,—a kind of a whitlow. A very small amount of decomposed pulp shut in at the apex, as is often the case, will in time cause irritation, leading to thickening of tissue around the apex of the tooth ; afterwards the part of the thickened peridental membrane and adjacent tissues about the apex of the root, will break down and then there is a sac. This sac is nature's barricade to protect the system from blood-poison.

Common sense suggests that the treatment of such cases should be to get at that irritating substance, whatever it may be, and let it out. The old practice was to enlarge the foramen of the root, and treat it through this hole until it was cured. We might as well attempt to pour water into a bottle that is already full, as to try to force medicine into a sac already full, unless the foramen is enlarged considerably; but this drilling the foramen larger is liable to form a sharp edge around the hole. To drill this sharp edge, Dr. Atkinson used a small excavator. It is a poor plan.

Nature is our best guide in these matters. She makes a fistulous drainage from the socket. Drilling through the gums and socket, to the interior of the trouble, is the only scientific plan of treatment of a case containing pus. I derived so much benefit from this treatment that in 1878 I gave a course of lectures on it, at two colleges. If any persons are sufficiently interested in the results of my labors in this line, they will find them published in the *Dental Cosmos* for July, August, September, and October, 1878; and in the *Missouri Dental Journal* for April, May, June, July,

August, September, and October, 1879. These eleven papers contain a great deal of experimental detail.

The ragged bone that the essayist speaks of is generally necrosed bone. After an abscess has found vent through the side of the socket, the sac changes in its character. It hardens, in a measure disappears, and if the alveolar process behind the sac is deprived of its nutrient supplies it dies, and the sac dies in consequence, leaving a chamber filled with liquid substance. I call these cases *sepulchral* abscesses. The proper treatment, of course, is to go in there and cut all the dead part out; cut with a sharp bur until the healthy tissue is reached; sometimes, however, it is difficult, if not impossible, to get all the dead bone out. In such instances there may be a temporary relief. Even if there is left some carious bone, it may, after a time, die,—become necrosed.

Sometimes the end of the root projecting into this cavernous place itself takes on a degenerative condition. In fact, often the ends of these roots are rotten. Those cases formerly plagued me very much. Once I had a case that was favorable to amputate the root, and I did so. Of course that left the opening into the pulp-chamber enlarged, so that it had to be filled with gold wire to make it tight; but the process cured the abscess, and since that time the amputation of roots has, with me, been a common practice. I published this operation, among others, in the *Dental Cosmos*, March, 1884.

Concerning creosote as a remedy, I look upon it in this way: if a sore is on the outside, an ulcer, we would not use creosote, nor would we use creosote in a flesh abscess just under the surface; we might perhaps wash it out with something mild, such as castile soap and water, but with bone abscesses, we are dealing with something different. Iodine preparations are disagreeable because they discolor, but they otherwise do very well. Regarding the cauterization of the interior of the sac, I do not quite agree with the essayist. I think it is proper to cauterize the interior of a sac. Of course there is no such thing as a "pyogenic membrane," but there is a sluggish surface which should be removed. When this is destroyed, and the parts are treated with some simple liquid, such as table-salt and water, injected every day or two, until that degenerated substance is all driven out, and the contents are prevented from becoming rancid by continuing injections of salt and water, Labarraque's solution, or wood creosote and alcohol (which is non-escharotic) once in five or seven days, it will generally be followed by healthy granulations and a cure. For cauterization of a sac I

have found escharotic creosote (coal tar creosote), or sulphate of zinc, as effective as anything. Either pump it through the canal of the tooth until it appears at the mouth of the fistula, or inject it through the fistula with a syringe.

*Dr. Darby*, of Philadelphia.—I have been much interested in the paper just read, but I do not know that I am prepared to discuss it. There are, however, a few points upon which I may say a few words.

The essayist spoke disparagingly of the use of creosote and carbolic acid. These are medicines which have been used in dentistry from time immemorial. Especially is this true of creosote, and, although it is not absolutely essential in a dental practice, it certainly has served, and is still serving, a very good purpose in the treatment of devitalized teeth and chronic alveolar abscess. We all know that many a sac at the apex of a root has been broken up and a fistula healed by the escharotic effect of good creosote. The same may be said of carbolic acid. There may be better remedies than either of these, still I am not yet prepared to abandon altogether good English creosote made by Squores, of London.

The essayist spoke in high terms of hot air for drying the canals of teeth. But, to be effective, it must have pressure behind it to force it into the root of the tooth. Some years ago I put into my laboratory an apparatus for giving me compressed air. It consists of the following:

A cylinder, made of galvanized iron, holding one hundred gallons, with a pipe running to my office, and at its end a gauge to denote the number of pounds pressure. A stop-cock within reach of the chair and a rubber tubing reaching to the mouth of the patient, with a Taft hot-air syringe, and a little spring cut off, to be held in the hand. In the laboratory, in close proximity to the cylinder, is an air-pump, and each morning the man pumps into this cylinder compressed air, anywhere from five to thirty pounds. By simply holding the bulb of my Taft hot-air syringe in the flame of my small Benson burner, I can have any degree of heat desired, and sufficient pressure back of it to force it into the root of the tooth.

To me this apparatus has been almost invaluable, as I am enabled to dry a tooth more thoroughly than I have ever been able to do in any other way.

I would indorse all that the essayist has said about the free use of alcohol for cleansing the root, but much prefer the absolute alcohol to the ninety-five per cent., which is commonly used.

Whenever opening a devitalized tooth, in which I suspect septic conditions, my practice has been to put into the pulp-chamber a few crystals of the permanganate of potassium, and then washing it with a solution of the same. After which I dry it with the hot-air blast. Then wash it with the absolute alcohol, and then dry it again, this time with as much heat as the patient will endure.

In regard to the substance which shall be used for filling the canal, I do not think there is much choice between several. The point is to get something into it that will thoroughly seal it.

Cotton has been recommended, and in Philadelphia there are some who must believe in it, for they certainly use it a great deal. Dr. Farrar has said that cotton decomposes. I am not an advocate of this method of filling roots, but candor compels me to say that I have often removed cotton from the roots of teeth years after it had been put there, and the roots seemed in a healthy condition and the cotton was not decomposed.

But there are better materials than cotton. If gold could be carried to the apex of the root in all instances, I would prefer to put it there, and fill the balance of the canal with oxychloride of zinc. This, in my opinion, is the ideal filling for the roots of teeth. It is antiseptic, is plastic, and, if well introduced, makes a perfect sealing of the tooth. Teeth that have been thoroughly cleansed, and then thoroughly filled with oxychloride of zinc, seldom have an unpleasant odor when opened.

I agree with the essayist in his belief that drilling through the end of the root for relief in acute abscess is not often attended with pleasing result. My own experience and observation teaches me that the practice is a bad one, and leads to difficulty when it becomes desirable to find the root. But I must take issue with him in the other surgical operation which he so highly commends,—namely, cutting or drilling through the alveolar process. This may do very well for the six anterior teeth, or for teeth with a single root, where the length can be ascertained, but when the abscess is at the apex of a double- or triple-rooted tooth, the operation is too uncertain.

*Dr. G. Lenox Curtis.*—Perhaps the best way to present my views of this paper will be to describe my own method of dealing with the class of cases it describes. This description has necessarily, owing to a pressure of other duties and the brief interval permitted me for preparation, been hastily and perhaps imperfectly thrown together.

The important thing to consider, when a devitalized tooth presents, is how to prevent the formation of an abscess, if that has not already occurred. Right here judgment and ability on the part of the dentist are required, and the manner in which he acquits himself will show the stuff of which he is made. Unless he lays the foundation in correct principle and skilful manipulation, the superstructure, of after treatment, will fall.

The first step is the thorough removal of the entire pulp *débris*, without which it is impossible to fill the canal properly. The opening up of the canal is best done in most cases with the flexible engine drill. There are comparatively few roots which cannot be opened up by this means to the very end; in the few which cannot, a fine flexible probe of piano wire, lubricated with a drop or two of carbolic acid, can be passed, with a little force, nearly, if not quite, to the apical foramen. The only exceptions to the rule to open the canal its entire length are those where the root is extremely crooked, rendering perforation likely, and those in which calcification has taken place. In the young the root-canals are much larger than in older persons, and there is, therefore, greater necessity for opening the canal freely. Too many operators make the fatal mistake of not cutting away sufficient of the tooth to allow the drilling to be done on a line parallel to the direction of the root.

The next step is the perfect cleansing of the canal. Simply forcing the ground-up dentine out by means of an air or water syringe, and then swabbing with cotton pushed into the canal as far as the probe usually employed will carry it, will not answer. That is not perfect cleansing, and is far different from what I mean. Too few dentists, I am afraid, have the proper instruments for accomplishing what I wish understood by perfect cleanliness, which is a task requiring time and patience.

My method is, first, to prevent, as far as possible, the entrance of moisture into the canal during the drilling; then, having desiccated the powdered bone with a blast of hot, dry air, I work into the canal with a rotary movement a delicate, tapering probe, frequently withdrawing it to carry out the power adhering to its sides. This procedure is continued till the powder is completely removed. The hot blast is then again applied until the patient complains of discomfort from the heat, when Darby paper-points are used to ascertain if there is any moisture remaining at the extremity of the canal. Sometimes it will occur that serum or blood oozes from the vessels at the end of the root, and finds its way back into the canal, a fact which can be only ascertained by the measure just noted, unless the exudation is considerable. (When the apical foramen is large, or the root not fully developed, as in the teeth of young persons, it is difficult to check the extravasation of blood, requiring delicate surgical treatment to heal the wound before the filling of the canal should be attempted. When the filling is inserted, precaution must be taken against too much pressure upon the freshly-healed surface, or the wound may reopen and complications follow.) With the tooth dried as far as the heat will penetrate, the remaining moisture in the canaliculi is absorbed, and the canal is ready for filling, unless the pulp is putrescent, the treatment in which case will be stated further on.

For filling root-canals I have tried cotton, gold, amalgam, and cement, and long since discarded them, because, in my judgment, it was not possible to do perfect work with them. Not one of those mentioned can be made to enter and fill the canaliculi, which is an important part of the operation, as lessening the possibilities of the decomposition of the animal matter of the dentine. Nor can any one of them, in my judgment, be perfectly adapted to the walls, with the possible exception of cases where the roots are straight and short, and can be freely reamed out. I therefore use for filling canals that which it seems to me has fewer objections and more desirable properties than anything else known to me,—namely, chlora-percha.

My method of introducing the chlora-percha is to flood the canal with chloroform, and, placing the patient in a position which will assist in the expulsion of air from the tooth, work the chloroform with a delicate probe to the end of the root. The thorough diffusion of the chloroform is, I believe, greatly assisted by capillary attraction, owing to the extreme dryness of the tooth. This is immediately followed by the introduction of gutta-percha canal-points, which, by the aid of a to-and-fro motion, are dissolved in the chloroform along their entire length, forming the chlora-percha, which is distributed into every portion of the interior of the root accessible. Point after point is forced in, and placed in this manner, until the entire canal and the open canaliculi are filled with the chlora-percha.

I employ this method in all devitalized teeth, providing, of course, where the pulp is putrescent, for its deodorization and sterilization. This, however, is a simple task; hot water and hot air are usually sufficient, few, if any, medicaments being required. Where pericemental inflammation exists, I prefer the tincture of iodine to any other dressing, though some of the derivatives of this drug, as iodoform, aristol, etc., will accomplish perhaps equally good results by longer application. I see no necessity for the use of carbolic acid or creosote in the treatment of this condition, except to avoid a greater and more obnoxious odor.

I am opposed to treating alveolar abscess in any other way than by a positive and accurate procedure,—amputating the root, etc.

For a number of years I relied entirely upon therapeutic measures in this trouble, among them the injection of carbolic acid and creosote, although I employed the latter very rarely, partly because of its objectionable odor and partly because of my belief that carbolic acid was better. It was generally necessary to enlarge the apical foramen and force the medicine through, in the hope that its escharotic effect would destroy the sac and fistula, and thus the disease eventually disappear. Usually this method consumed much time, and only those that could well afford the luxury of such treatment received it. The greatest care is required in the use of carbolic acid lest it should accidentally come in contact with the gum. I have not infrequently observed much damage to the alveolus following its employment.

Early in my practice I realized the importance of positive surgical treatment in alveolar abscess, and for eight years I have employed that method almost exclusively, and, I am glad to say, with almost universal success.

After the root has been cleansed and filled as before described, the next step is to complete the filling of the cavity in the tooth. This should be a permanent operation, as the tooth is never again to be opened. A local or general anæsthetic is now resorted to, as the patient or the case requires. If the fistula exists, the probe usually leads directly into the sac or the excavation in the alveolus, which is invariably present. If there is no fistula, or if it is at some distance from the seat of trouble, a direct opening into the sac must be made. For this purpose I prefer a spear-pointed drill, which is thrust through the gum and alveolus in a direct line with the root. It is simply a matter of practice and delicacy of touch to know where to drill and when to stop. A rose bur of suitable size is now inserted and rapidly revolved, to cut away the sac and necrosed bone. If the apex of the root is necrosed, it is likewise burred away, or if the necrosis is extensive, so much of the root is amputated as is necessary.

Amputation usually requires a larger opening through the wall of the alveolus, and in extreme cases a flap of the gum and alveolus is turned back to allow room for the saw and for the removal of the diseased tissue. Then cleanse the cavity and check the hemorrhage.

Hot-water injections will usually stop the bleeding, when an opportunity is afforded for ocular examination. Sterilize the wound, and if it is large, pack with antiseptic lint, continuing the dressing until no longer necessary. The antiseptic dressing may consist of injections of suitable solutions to aid in the healing of the parts.

If the operation has been inconsiderable, the wound may be left entirely alone after injecting with hot water and removing the *débris*, with the expectation of a cure. Even a fistula at some distance, necessitating a false opening into the sac, requires little or no treatment and soon disappears.

In the large majority of cases the amputation can be accomplished at the time the root is filled, but to impress upon you the importance of immediately curing all classes of abscess with fistulous opening, it is only necessary to remind you that wherever found they are usually tubercular. If the antrum or nares is penetrated by a fistula, special treatment is commonly necessary.

I believe that in no case should the root-canal be left open, because so long as it remains open, resolution at the end of the root is practically impossible, owing to septic influence at the base of the newly-formed tissue, which again breaks down, unless the risk is provided against by immediate filling. To enlarge the apical foramen only complicates matters, by rendering more difficult the adaptation of a perfect filling, besides endangering the integrity of the new tissue by the likelihood of the filling-material being forced beyond the root apex.

The operation of alveolotomy is to my mind based upon a rational view of health and disease. An abscess is nature's way of getting rid of a disturbing influence, and often considerable damage to the surrounding parts and great suffering to the patient are the outcome of her efforts to dislodge the intruder. The indication is, therefore, to assist nature by removing the affected tissue which cannot be restored to health and usefulness. My vote is therefore for surgical procedures in both acute and chronic alveolar abscess,—some differences in the care of the two forms are required,—because experience has taught me that it is the best and quickest method to assist nature to assert herself.

And now allow me a suggestion or two as to why so many teeth remain abscessed. Is it due to lack of knowledge as to what the disease is and how to find it? Is it because the practice of the average dentist is too full of the ordinary work to allow sufficient time to treat these cases when they are found? Is it because he does not know just what to do, how to do, and when to do? Does the general office work keep him from special study and accomplishment in the direction of surgery? Has he not sufficient confidence in the honor of some dentist of his acquaintance whom he knows to be successful in this line of work to warrant his sending patients to that expert for treatment in his specialty, with the full expectation that operations coming within the province of the general practitioner's accomplishments will not be attempted? Is there danger in such a course of loss of the patient, or of uncharitable criticism of the dentist who does not do surgical operations or of his work?

It seems to me that each of these conditions exists to some extent, and that their existence tends to retard progress, to prevent our doing our full duty towards those who confide in us, and who expect from us the best that knowledge and skill can accomplish. This is not as it should be, and I think I see the dawn of a better day.

As has already occurred in medicine and surgery, the time has come for the establishment of specialties and special practitioners in dentistry; when men adapted by training or temperament to a particular branch of practice shall consecrate themselves and their energies to that field. Certainly one who with the proper foundation does so devote himself to a special phase of practice should be most capable of judging of the necessities for specific operations, and be best calculated to perform them. To me it is a pleasure to feel that this happy state of affairs is just before us, and I have no hesitation in predicting that ten years from now there will be comparatively few general practitioners of dentistry.

*Dr. Crouse*, of Chicago.—When I came into the room and remained for about five minutes, I had a sensation that I was probably in a kindergarten; and then my attention changed to something else, and the same sensation came over me again when the next speaker took his place, and now, Mr. President, I do not know just how I do feel.

I have heard the discussion of alveolar abscesses, although I have not read the paper. I tried to be in two places at once, and tried to hear two papers, one here and the other at the lower part of the building, and therefore did not get either. Yet I am delighted to speak on this subject, which I suppose is the treatment of alveolar abscesses.

I would like to take up the last speaker first, because I might forget something in his argument. These surgical dentists, these men who get surgery chronic in their minds, think that the only way of doing anything is by a surgical operation. If there is any place I would steer my patients clear of, it is the office of one of these surgeons,—the one who thinks his work is to cut and slash all the time.

The trouble with surgical dentists is that they work at the wrong end. I have treated a great number of alveolar abscesses, and I have never found but three which did not get well with the treatment I will describe.

I happen to be in a field of colleges always productive of a great deal of surgery. We have five or six colleges in active operation. Do you not pity the patients when they have to submit to so many surgical operations a day? A law prohibiting dental surgeons or surgical dentists from operating, I think, would be a good thing. It might be a hardship to those who want to practise, but it would be a great blessing to the dear people.

What is the treatment I advocate? It is a simple matter, especially simple since we have the rubber-dam, and can dry out the tooth and prevent the medicine from coming in contact with the tissues. What is the operation? Prepare your cavity; it is not necessary to give the details, except that care must be taken not to force the broach into the pulp-canal, or get the cavity clogged with foreign matter; take a piece of soft india-rubber, cut it as near the size and shape of the cavity as you can; fill the cavity with carbolic acid, place the india-rubber into the cavity, and with it force the carbolic acid out through the fistulous opening. This is readily done with a blunt instrument and sudden force against the rubber, such force as is used in packing gold by hand-pressure. In my hands this has been the most effectual way of accomplishing the treatment. One such treatment is generally sufficient.

The frequent treatments described by the former speaker as being a luxury I am inclined to think my patients would not enjoy. I may be mistaken, and if I thought they wanted the pleasure of coming often and going through all that surgery, I do not know but I could work myself up to it.

Dr. Darby spoke of a class of cases where the pulp died under an oxychloride cap, and the root was found to be dry. In such cases I think the ends of the roots become encysted, and it is better to leave them alone than to treat them.

I do not like to speak on a paper that I have not heard, and if there are any points that I have not hit, I will be glad to know what they are.

*Dr. Curtis.*—I would like to hear some one challenge Dr. Crouse's views. I fancy he was joking.

*Dr. Crouse.*—For fear some one will so regard it, I will say that I am perfectly in earnest.

*Dr. Van Woert.*—In my preparation for the discussion of this paper, I was obliged to confine myself to the small amount of data contained in the short synopsis given in the regular notice. Therefore it will not be as complete as I should like to have it. In the first place, the notice says that the essayist will define his reasons for not using carbolic acid in the treatment of pulpless teeth and alveolar abscess. In my opinion it makes very little difference what theory may be advanced for not using carbolic acid; the fact remains that when you want to be absolutely certain, and count on results, you must accept the inevitable, and fall back on this old, reliable agent.

We are all ready to hail with delight anything that may possibly do away with the use of this offensive drug, but as yet there has been nothing presented, to my knowledge, that can take its place. Carbolic acid has made a reputation for itself that I fear it will be hard to kill. My experience with the substitutes offered, so far, has been anything but satisfactory, and I am confident that Dr. Porter will regret it if he abandons its use.

Second. The objection to leaving nerve-canals open in all cases where a fistula exists. When this is the case, I can see no reason for leaving the root-canal open. After freeing it from the septic matter with which it is clogged, it is my practice to fill at once.

Third. I am a strong advocate of immediate root-filling in all cases, for the following reasons: When a case of apical pericementitis presents itself, or any of the difficulties following, it is plain that such is the result of one of two causes,—either mechanical injury or the presence of septic matter in the roots. If the latter, by removing the cause and restoring the parts to a thoroughly aseptic condition, nature, in ninety-nine out of a hundred cases, will take up the fight and set matters straight without the aid of surgery or medicaments.

Fourth. The enlargement of the apical foramen I consider, as a rule, a bad practice, but there are cases where it is permissible and even advisable. The extraction and replanting of the tooth is at times really necessary; for instance, in excessive curvatures or malformations of the roots.

Fifth. I am a little surprised at his reasons for performing alveolotomy. I did not suppose there were any in this advanced age of surgery who would wait for the disease to produce a fistulous opening. I thought it was the practice to operate at once when cases of this kind presented themselves. Of course, the kind of operation depends entirely upon the state of advancement the disease is in when presented. The operation of alveolotomy would seem to me unnecessary in most cases.

As I purpose following these remarks with my treatment, it will be a very easy matter to see why I take this stand.

Sixth. I fail to quite understand his reasons for surgical treatment in chronic abscess after the canal has been filled. If he means that no medicament whatever is to be used, I cannot agree with him. On the contrary, should he mean that by gaining access for evacuating the sac by surgical means, after which medicaments are used for the treatment of the case, I fully concur with him.

Now, in vindication of what I have just said, I give the following as my reasons for using carbolic acid and the objections taken to the points raised by Dr. Porter.

I think if the gentlemen who have talked of the different preparations for filling roots will try out of the mouth the experiments that I have suggested on several occasions, such as oxychloride, chlora-percha, gutta-percha, oxyphosphate, or gold, and then immerse the tooth in an aniline dye for five or six hours, after which thoroughly dry, then, by splitting them open, it will be found that the entire length of the root-canal is thoroughly stained. This will be found true of any substance that changes after having been placed in position. Therefore I do not consider any of them proper materials for this operation. I want something in the canal that will remain just as I place it; and I am happy to say I have such a preparation in my office, which is giving great satisfaction to a number of the members of our profession as well as myself.

The originator of the compound, as I understand, was Dr. Bronson, who gave it to Dr. Lord; and it was through Drs. Lord and Parker that I first learned of it.

Originally it was a combination of iodoform, zinc oxide, and vaseline, and, to do away with the disagreeable odor, I substituted iodol for the iodoform; and later on I changed the proportiōns and added to it oil of cinnamon. The following is the formula as I now use it:

Iodol, gr. x;
Zinci oxidi, gr. xx;
Ol. cinnamomi, gt. v;
Vaseline carbol., q. s.

Mix at a temperature of 140° F., to form a stiff paste.

This paste is so thick and dense that it can be rolled into shape very similar to the gutta-percha points manufactured for filling root-canals. It is immaterial what the temperature of the surrounding parts may be, you cannot get inflammation enough to cause the least change of the vaseline, a difficulty which I had in the beginning from macerating the mixture cold.

And here I would add another very useful remedy to this; one that I find of great service in acute cases of pericementitis: One part tr. capsicum to two parts vin. opii.

This, applied on absorbent cotton, a small piece of felt, or blotting paper, will relieve pain quicker than any local application I have ever tried.

And finally, let me add one more word in favor of carbolic acid. In my opinion, sustained by a long experience, carbolic acid is the one of all drugs the results from which you can depend upon. There has been a number of theories advanced for discarding it, one of which is the coagulation of albumen in the roots; and yet, notwithstanding this, the effect is satisfactory; and until we can prove all that is brought against it, we had best stand by it as an old friend. I sincerely hope that this paper will not influence any of the younger members of the profession to discard so valuable an agent.

On motion, a vote of thanks was tendered to the essayist and the gentlemen who discussed the paper.

Adjourned.

JOHN I. HART, D.D.S.,
*Editor New York Odontological Society.*

